# Potassium trifluoro­[(*Z*)-3-methoxy­prop-1-en­yl]borate

**DOI:** 10.1107/S1600536808036428

**Published:** 2008-11-13

**Authors:** Julio Zukerman-Schpector, Rafael C. Guadagnin, Hélio A. Stefani, Lorenzo do Canto Visentin

**Affiliations:** a Department of Chemistry, Universidade Federal de São Carlos, 13565-905 São Carlos, SP, Brazil; b Departamento de Farmácia, Faculdade de Ciências Farmacêuticas, Universidade de São Paulo, São Paulo, SP, Brazil; c Instituto de Química, Universidade Federal do Rio de Janeiro, RJ, Brazil

## Abstract

In the title salt, K^+^·C_4_H_7_BF_3_O^−^, the K atom is surrounded by six anions making close contacts through seven F [K⋯F = 2.779 (1)–3.048 (1) Å] and two O [K⋯O = 2.953 (2) and 3.127 (2) Å] atoms in a trivacant *fac*-vIC-9 icosa­hedral coordination geometry.

## Related literature

For related structures, see: Caracelli *et al.* (2007 ▶); Stefani *et al.* (2006 ▶); For related literature, see: Ruiz-Martínez *et al.* (2008 ▶); Vieira *et al.* (2008 ▶).
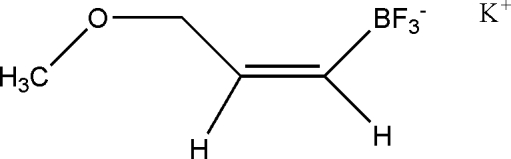


## Experimental

### 

#### Crystal data

K^+^·C_4_H_7_BF_3_O^−^
*M* *_r_* = 178.01
Monoclinic, 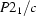

*a* = 10.882 (2) Å
*b* = 7.2668 (15) Å
*c* = 9.2317 (18) Å
β = 101.52 (3)°
*V* = 715.3 (3) Å^3^
*Z* = 4
Mo *K*α radiation
μ = 0.72 mm^−1^
*T* = 291 (2) K
0.31 × 0.22 × 0.11 mm

#### Data collection

Nonius KappaCCD diffractometer
Absorption correction: multi-scan (*SADABS*; Bruker, 2006 ▶) *T*_min_ = 0.804, *T*_max_ = 0.924
16605 measured reflections
1327 independent reflections
1182 reflections with *I* > 2σ(*I*)
*R*_int_ = 0.059

#### Refinement

*R*[*F* ^2^ > 2σ(*F* ^2^)] = 0.027
*wR*(*F* ^2^) = 0.072
*S* = 1.00
1327 reflections
92 parameters
H-atom parameters constrained
Δρ_max_ = 0.25 e Å^−3^
Δρ_min_ = −0.20 e Å^−3^

### 

Data collection: *COLLECT* (Nonius, 1998 ▶); cell refinement: *PHICHI* (Duisenberg *et al.*, 2000 ▶); data reduction: *EVAL-14* (CCD) (Duisenberg *et al.*, 2003 ▶); program(s) used to solve structure: *SIR97* (Altomare *et al.*, 1999 ▶); program(s) used to refine structure: *SHELXL97* (Sheldrick, 2008 ▶); molecular graphics: *ORTEP-3 for Windows* (Farrugia, 1997 ▶); software used to prepare material for publication: *WinGX* (Farrugia, 1999 ▶).

## Supplementary Material

Crystal structure: contains datablocks global, I. DOI: 10.1107/S1600536808036428/ng2510sup1.cif

Structure factors: contains datablocks I. DOI: 10.1107/S1600536808036428/ng2510Isup2.hkl

Additional supplementary materials:  crystallographic information; 3D view; checkCIF report

## Figures and Tables

**Fig. 1.**
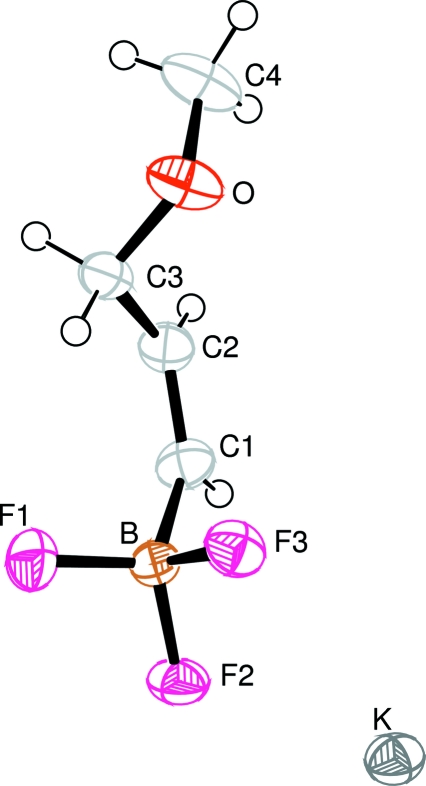
The molecular structure of the title compound showing atom labelling scheme and displacement ellipsoids at the 50% probability level (arbitrary spheres for the H atoms).

**Fig. 2.**
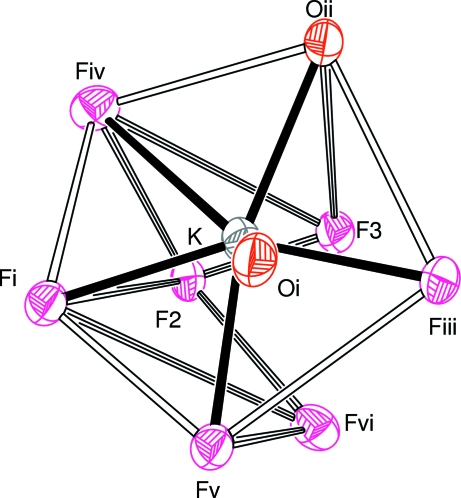
The trivacant icosahedron, fac-vIC-9, around the K+ ion. Symmetry operations: i = 1 - x, y - 1/2, 1/2 - z; ii = 1 - x, y - 1, -z; iii = 1 - x, y + 1/2, 1/2 - z; iv = 1 - x, -2 - y, -z; v = x, -3/2 - y, z - 1/2; vi = x, -3/2 - y, 1/2 + z.

## supplementary crystallographic information

### Comment

Organic compounds of tellurium, such as *Z*-vinylic tellurides, are important synthetic precursors of organometallic molecules and organic salts and can be useful in the synthesis of new potassium vinyl trifluoroborate salts. Organotrifluoroborates represent an alternative to boronic acids, boronate esters, and organoboranes for use in the Suzuki-Miyaura reaction and other transition-metal-catalyzed cross-coupling reactions (Vieira *et al.* 2008). Following the ideas of Ruiz-Martínez *et al.* (2008) the geometry around the K^+^ ion can be described as a trivacant icosahedron, *fac*-vIC-9, a non spherical shape, as shown in Figure 2. The independent molecules in (I) are connected *via* C3···F2^i^ = 3.214 (2) Å, C3—H3B···F2^i^ = 137° (i = *x* - 1/2, -*y* + 3/2, *z*).

### Experimental

nBuLi (0.8 mmol) was added dropwise at 203 K to a solution of the appropriated *Z*-vinylic telluride (1 mmol) in Et_2_O (6 ml). The bath temperature was raised to 253 K. After 20 minutes B(OiPr)_3_ (1.0 mmol) was added at 233 K. After 1 h, a aqueous solution of KHF_2_ (4 mmol in 10 ml of water) was added to the reaction mixture. Then, the solvent and water were eliminated by evaporation. To the obtained solid hot acetone was added and the bulk reactional was filtered and dried, yielding 67% of (*Z*)-potassium vinyltrifluoroborate salt. Single crystals were obtained by slow evaporation from Et_2_O.

### Refinement

The H atoms were refined in the riding-model approximation, with C—H = 0.93–0.97Å and *U*_iso_(H) = 1.5*U*_eq_(methyl C) or 1.2*U*_eq_(remaining C).

### Crystal data

**Table:**

K^+^·C_4_H_7_BF_3_O^–^	*F*_000_ = 360
*M**_r_* = 178.01	*D*_x_ = 1.653 Mg m^−^^3^
Monoclinic, *P*2_1_/*c*	Mo *K*α radiation λ = 0.71073 Å
Hall symbol: -P 2ybc	Cell parameters from 9536 reflections
*a* = 10.882 (2) Å	θ = 2.3–21.8º
*b* = 7.2668 (15) Å	µ = 0.72 mm^−^^1^
*c* = 9.2317 (18) Å	*T* = 291 (2) K
β = 101.52 (3)º	Block, colourless
*V* = 715.3 (3) Å^3^	0.31 × 0.22 × 0.11 mm
*Z* = 4	

### Data collection

**Table:**

Enraf–Nonius KappaCCD diffractometer	1327 independent reflections
Radiation source: fine-focus sealed tube	1182 reflections with *I* > 2σ(*I*)
Monochromator: graphite	*R*_int_ = 0.059
*T* = 290(2) K	θ_max_ = 25.5º
φ and ω scans	θ_min_ = 4.3º
Absorption correction: multi-scan(SADABS; Bruker, 2006)	*h* = −13→13
*T*_min_ = 0.804, *T*_max_ = 0.924	*k* = −8→8
16605 measured reflections	*l* = −11→11

### Refinement

**Table:**

Refinement on *F*^2^	Secondary atom site location: difference Fourier map
Least-squares matrix: full	Hydrogen site location: inferred from neighbouring sites
*R*[*F*^2^ > 2σ(*F*^2^)] = 0.027	H-atom parameters constrained
*wR*(*F*^2^) = 0.072	*w* = 1/[σ^2^(*F*_o_^2^) + (0.0364*P*)^2^ + 0.2723*P*] where *P* = (*F*_o_^2^ + 2*F*_c_^2^)/3
*S* = 1.00	(Δ/σ)_max_ < 0.001
1327 reflections	Δρ_max_ = 0.25 e Å^−^^3^
92 parameters	Δρ_min_ = −0.20 e Å^−^^3^
Primary atom site location: structure-invariant direct methods	Extinction correction: none

### Special details

**Table:**

Geometry. All e.s.d.'s (except the e.s.d. in the dihedral angle between two l.s. planes) are estimated using the full covariance matrix. The cell e.s.d.'s are taken into account individually in the estimation of e.s.d.'s in distances, angles and torsion angles; correlations between e.s.d.'s in cell parameters are only used when they are defined by crystal symmetry. An approximate (isotropic) treatment of cell e.s.d.'s is used for estimating e.s.d.'s involving l.s. planes.
Refinement. Refinement of *F*^2^ against ALL reflections. The weighted *R*-factor *wR* and goodness of fit *S* are based on *F*^2^, conventional *R*-factors *R* are based on *F*, with *F* set to zero for negative *F*^2^. The threshold expression of *F*^2^ > σ(*F*^2^) is used only for calculating *R*-factors(gt) *etc*. and is not relevant to the choice of reflections for refinement. *R*-factors based on *F*^2^ are statistically about twice as large as those based on *F*, and *R*- factors based on ALL data will be even larger.

### Fractional atomic coordinates and isotropic or equivalent isotropic displacement parameters (Å^2^)

**Table:**

	*x*	*y*	*z*	*U*_iso_*/*U*_eq_	
B	0.64509 (18)	−0.7960 (2)	0.0837 (2)	0.0305 (4)	
C1	0.77652 (16)	−0.7044 (2)	0.16327 (19)	0.0371 (4)	
H1	0.8143	−0.7569	0.253	0.045*	
C2	0.83849 (15)	−0.5637 (2)	0.11809 (19)	0.0352 (4)	
H2	0.9123	−0.5254	0.1798	0.042*	
C3	0.79730 (17)	−0.4641 (2)	−0.02438 (18)	0.0379 (4)	
H3A	0.863	−0.4722	−0.0814	0.045*	
H3B	0.7233	−0.5242	−0.0806	0.045*	
C4	0.8776 (2)	−0.1624 (3)	0.0361 (3)	0.0588 (6)	
H4A	0.8539	−0.0349	0.0304	0.088*	
H4B	0.9337	−0.1857	−0.0299	0.088*	
H4C	0.9186	−0.1916	0.1354	0.088*	
F1	0.63816 (10)	−0.84405 (14)	−0.06632 (11)	0.0438 (3)	
F2	0.61847 (9)	−0.95573 (12)	0.16043 (10)	0.0396 (3)	
F3	0.54238 (9)	−0.67152 (13)	0.08481 (11)	0.0384 (3)	
K	0.40701 (3)	−0.83116 (5)	0.27917 (4)	0.03619 (15)	
O	0.76899 (12)	−0.27319 (17)	−0.00483 (16)	0.0469 (3)	

### Atomic displacement parameters (Å^2^)

**Table:**

	*U*^11^	*U*^22^	*U*^33^	*U*^12^	*U*^13^	*U*^23^
B	0.0374 (9)	0.0264 (9)	0.0288 (9)	−0.0009 (7)	0.0095 (7)	0.0035 (7)
C1	0.0362 (9)	0.0389 (9)	0.0344 (8)	−0.0009 (7)	0.0030 (7)	0.0085 (7)
C2	0.0304 (8)	0.0362 (9)	0.0376 (9)	−0.0027 (7)	0.0034 (7)	0.0001 (7)
C3	0.0488 (10)	0.0295 (9)	0.0353 (9)	−0.0089 (7)	0.0080 (7)	−0.0018 (7)
C4	0.0504 (12)	0.0335 (10)	0.0867 (17)	−0.0095 (9)	−0.0005 (11)	−0.0073 (10)
F1	0.0508 (6)	0.0504 (6)	0.0312 (5)	−0.0058 (5)	0.0104 (4)	−0.0054 (4)
F2	0.0477 (6)	0.0291 (5)	0.0412 (6)	−0.0057 (4)	0.0064 (4)	0.0077 (4)
F3	0.0359 (5)	0.0332 (5)	0.0454 (6)	0.0032 (4)	0.0059 (4)	0.0007 (4)
K	0.0380 (2)	0.0316 (2)	0.0396 (2)	0.00022 (14)	0.00919 (16)	0.00628 (15)
O	0.0390 (7)	0.0293 (6)	0.0682 (9)	−0.0014 (5)	0.0007 (6)	0.0024 (6)

### Geometric parameters (Å, °)

**Table:**

B—F1	1.415 (2)	C2—H2	0.9300
B—F2	1.4198 (19)	C3—O	1.440 (2)
B—F3	1.440 (2)	C3—H3A	0.9700
B—C1	1.615 (3)	C3—H3B	0.9700
B—K	3.450 (2)	C4—O	1.417 (2)
C1—C2	1.337 (2)	C4—H4A	0.9600
C1—H1	0.9300	C4—H4B	0.9600
C2—C3	1.490 (2)	C4—H4C	0.9600
			
F1—B—F2	108.08 (13)	O—C3—H3A	109.0
F1—B—F3	105.82 (13)	C2—C3—H3A	109.0
F2—B—F3	105.89 (13)	O—C3—H3B	109.0
F1—B—C1	114.59 (15)	C2—C3—H3B	109.0
F2—B—C1	111.11 (13)	H3A—C3—H3B	107.8
F3—B—C1	110.86 (13)	O—C4—H4A	109.5
C2—C1—B	129.00 (15)	O—C4—H4B	109.5
C2—C1—H1	115.5	H4A—C4—H4B	109.5
B—C1—H1	115.5	O—C4—H4C	109.5
C1—C2—C3	124.44 (15)	H4A—C4—H4C	109.5
C1—C2—H2	117.8	H4B—C4—H4C	109.5
C3—C2—H2	117.8	C4—O—C3	113.13 (14)
O—C3—C2	113.05 (14)		
			
F1—B—C1—C2	50.8 (3)	B—C1—C2—C3	−2.2 (3)
F2—B—C1—C2	173.64 (17)	C1—C2—C3—O	116.83 (19)
F3—B—C1—C2	−68.9 (2)	C2—C3—O—C4	76.3 (2)

## References

[bb1] Altomare, A., Burla, M. C., Camalli, M., Cascarano, G. L., Giacovazzo, C., Guagliardi, A., Moliterni, A. G. G., Polidori, G. & Spagna, R. (1999). *J. Appl. Cryst.* **32**, 115–119.

[bb2] Bruker (2006). *SADABS* Bruker AXS Inc., Madison, Wisconsin, USA.

[bb3] Caracelli, I., Stefani, H. A., Vieira, A. S., Machado, M. M. P. & Zukerman-Schpector, J. (2007). *Z. Kristallogr. New Cryst. Struct.* **222**, 345–346.

[bb4] Duisenberg, A. J. M., Hooft, R. W. W., Schreurs, A. M. M. & Kroon, J. (2000). *J. Appl. Cryst.* **33**, 893–898.

[bb5] Duisenberg, A. J. M., Kroon-Batenburg, L. M. J. & Schreurs, A. M. M. (2003). *J. Appl. Cryst.* **36**, 220–229.

[bb6] Farrugia, L. J. (1997). *J. Appl. Cryst.* **30**, 565.

[bb7] Farrugia, L. J. (1999). *J. Appl. Cryst.* **32**, 837–838.

[bb8] Nonius (1998). *COLLECT* Nonius BV, Delft, The Netherlands.

[bb9] Ruiz-Martínez, A., Casanova, D. & Alvarez, S. (2008). *Dalton Trans.* pp. 2583–2591. 10.1039/b718821h 18443701

[bb10] Sheldrick, G. M. (2008). *Acta Cryst.* A**64**, 112–122. 10.1107/S0108767307043930 18156677

[bb11] Stefani, H. A., Cella, R., Zukerman-Schpector, J. & Caracelli, I. (2006). *Z. Kristallogr. New Cryst. Struct.* **221**, 167–168.

[bb12] Vieira, A. S., Fiorante, P. F., Zukerman-Schpector, J., Alves, D., Botteselle, G. V. & Stefani, H. A. (2008). *Tetrahedron*, **64**, 7234–7241.

